# Obesity and periodontal disease

**DOI:** 10.4103/0972-124X.70827

**Published:** 2010

**Authors:** Sunitha Jagannathachary, Dinesh Kamaraj

**Affiliations:** *Department of Periodontics, College of Dental Sciences, Davangere, Karnataka, India*; 1 *Department of Periodontics, Rajas Dental College, Vadakankulam, Tamil Nadu, India*

**Keywords:** Adipokines, obesity, periodontal disease

## Abstract

Obesity is characterized by the abnormal or excessive deposition of fat in the adipose tissue. Its consequences go far beyond adverse metabolic effects on health, causing an increase in oxidative stress, which leads not only to endothelial dysfunction but also to negative effects in relation to periodontitis, because of the increase in proinflammatory cytokines. Thus obesity appears to participate in the multifactorial phenomenon of causality of periodontitis through the increased production of reactive oxygen species. The possible causal relationship between obesity and periodontitis and potential underlying biological mechanisms remain to be established; however, the adipose tissue actively secretes a variety of cytokines and hormones that are involved in inflammatory processes, pointing toward similar pathways involved in the pathophysiology of obesity, periodontitis and related inflammatory diseases. So the aim of this article is to get an overview of the association between obesity and periodontitis and to review adipose-tissue – derived hormones and cytokines that are involved in inflammatory processes and their relationship to periodontitis.

## INTRODUCTION

Obesity, defined as a body mass index (BMI) >30.0 kg/m^2^, is a major public health problem today. The prevalence of obesity has increased substantially over the past decades in most industrialized countries. Obesity is a risk factor for several chronic diseases, most notably hypertension, type 2 diabetes, dyslipidemia and coronary heart disease.[1–3] Since adiposity can be considered a systemic disease that predisposes to a variety of comorbidities and complications that affect overall health, obese persons and all health professionals including dentists should require awareness regarding obesity. Further, recent studies have suggested that obesity is also associated with oral diseases, particularly periodontitis.[4–9] In fact, the adipose tissue secretes several cytokines and hormones that are involved in inflammatory processes, suggesting that similar pathways are involved in the pathophysiology of obesity and periodontitis.

## DEFINITION AND ASSESSMENT OF OBESITY

The definition of obesity is based on body mass index (BMI, also called Quetelet Index), which is the ratio of body weight (in kg) to body height (in m) squared.[1] BMI is highly correlated with fat mass and morbidity and mortality and therefore sufficiently reflects obesity-related disease risk in a wide range of populations; however, there are some limitations. For example, for the same BMI, older persons tend to have a higher body fat composition; and therefore, risk assessment by BMI is less accurate in older people (over 65 years of age).

Body fat distribution is assessed by the measurement of waist circumference, with 102 cm in men and 88 cm in women, respectively, being the cut-off point for abdominal obesity associated with an increased risk of morbidity.[1] Waist circumference shows a close correlation with the amount of visceral adipose tissue, and visceral adipose tissue has been shown to be metabolically more active and to secrete far greater amounts of cytokines and hormones compared with subcutaneous adipose tissue.[10] Recent large studies have indicated that measurement of waist circumference or waist-hip ratio may be a better disease risk predictor than BMI, and there is still intensive research ongoing as to whether BMI, waist circumference or both should be used to assess disease risk[11,12] [Table 1].

**Table 1:** Classification and definition of overweight and obesity (based on expert panel, 1998)

		Disease risk† relative to normal weight circumference and waist	
Classification	BMI (kg/m^2^)	Men≤102 cm Women≤88 cm	Men>102 cm Women>88 cm
Underweight	<18.5	—	—
Normal	18.5-24.9	—	—
Overweight	25.0-29.9	Increased	High
Obese - Class I	30.0-34.9	High	Very high
Obese - Class II	35.0-39.9	Very high	Very high
Obese - Class III	≥40	Extremely high	Extremely high

Established for non-Asian populations. The recently proposed classification for Asian population is BMI<18.5, underweight, 18.5-22.9, normal weight; 23.0-24.9, overweight 25.0-29.9, obese class I; ≥30.0 obese class II (WHO/IASO/IOTF, 2000).

† Disease rick for type 2 diabetes, hypertension, and cardiovascular\disease.

Several other diagnostic tools are available to assess body fat composition, such as measurement of (subcutaneous) skin fold by means of a caliper or ultrasound, bioelectrical impedance analysis (BIA), densitometry or imaging procedures (Computed tomography, Nuclear Magnetic Resonance); however, most of these procedures are not readily available in clinical practice and do not add substantial information for risk assessment in an individual, beyond BMI and waist circumference.

### Prevalence

Over the period 1960-1980, the prevalence of overweight and obesity among adults and of overweight among children, was relatively constant. Above 13% of adults were obese, and 5% of children were overweight. However, data from the National Health and Nutrition Examination Survey III (1989-1991) showed that obesity in adults and overweight in children had markedly increased in comparison to the previous survey. Those trends continued such that approximately 31% of American adults now meet the criteria for obesity. More than 65% of the United States adult population has a body mass index of ≥25 kg/m2, and 15.8% of children aged 6-11 years and 16.1% of adolescents aged 12-19 years are overweight. Thus in a relatively short time period, the prevalence of obesity among adults has doubled, and the prevalence of overweight among children and adolescents has tripled. In the year 2004, approximately 34.1% of the US population was overweight; and about 32.2%, obese.[1,7]

### Obesity-related diseases

#### Hypertension

Overweight and obesity have long been recognized as important determinants of elevated blood pressure levels. It is well established that weight gain is consistently associated with increased blood pressure, and that weight loss decreases blood pressure independent of changes in sodium intake. Compared with normal-weight individuals, obese persons have an up to 5 times higher risk of hypertension, and up to two thirds of cases of hypertension can be attributed to excess weight. Mechanisms that have been implicated in the development of obesity-related hypertension include increased sympathetic nerve activity, sodium and volume retention, renal abnormalities, insulin resistance, hyperleptinemia and increased secretion of angiotensinogen from adipocytes.[3,13]

#### Type 2 diabetes

The relationship between obesity and type 2 diabetes is particularly close. Obese persons have a more than 10-fold increased risk of developing type 2 diabetes compared with normal-weight persons. Type 2 diabetes develops due to an interaction between insulin resistance and beta cell failure. Several factors, including lipotoxicity and glucose toxicity, as well as obesity-derived cytokines, have been implicated in these processes.[8,14]

#### Cardiovascular disease

Obese persons have an about 1.5-fold increased risk for cardiovascular disease (including coronary heart disease and cerebrovascular disease), and between 10% and 15% of all cases of cardiovascular disease can be attributed to overweight and obesity. Obesity is also associated with an about 2-fold higher risk of heart failure and a 50% increased risk of atrial fibrillation.[10,15]

#### Osteoarthritis

Obesity is associated with bone knee and hip arthritis, and with arthritis involving the carpometacarpal joints of the hand. Recent studies have proved that being overweight antedates the development of knee osteoarthritis and increases the risk of radiographic progression.[16]

#### Respiratory disorders

Visceral fat accumulation results in restrictive respiratory function with reduced forced vital capacity and expiratory reserve volume. Obesity is the major reversible risk factor for obstructive sleep apnea syndrome. The prevalence rises from 2% to 4% in the general population to a prevalence of at least 40% in morbidly obese patients. Orofacial findings of this syndrome includes a retrognathic mandible, narrow palate, large neck circumference, long soft palate, tonsillar hypertrophy, nasal septal deviation and relative macroglossia. Waist circumference tends to be a better predictor of this syndrome than body mass index.[16,17]

#### Metabolic syndrome

The metabolic syndrome is a concept that encompasses metabolic abnormalities that co-occur to a greater degree than would be expected by chance alone, and which predisposes individuals at a high risk to develop cardiovascular disease. Although the exact underlying cause of metabolic syndrome is unknown, the more recent definitions emphasize the focus on abdominal obesity as its core component (International Diabetes Federation, 2005). This approach is supported by a growing number of studies showing that the adipose tissue itself is capable of producing several hormones and proteins, which are involved in the development of obesity-related diseases.[16,18]

## MORTALITY

Whether overweight and obesity affect disease prognosis and total mortality is an ongoing area of research, and recently published studies have found contradictory results. For example, although obesity increases the risk of heart failure, the studies found that among persons with prevalent heart failure, obese individuals are likely to have a better prognosis than non-obese individuals. This is likely due to the fact that lower BMI reflects wasting processes in this patient group (as in other chronic diseases). Further, several studies have found a U-shaped association between BMI and total mortality, with a minimum at a BMI of approximately 25.0 kg/m^2^,.[19] However, it has been argued that these analyses may be confounded by smoking (smokers are usually leaner than nonsmokers but have a higher risk of mortality) or underlying prevalent chronic diseases (individuals with chronic diseases often have lower body weight). Clearly, further studies are needed to examine the effect of obesity on morbidity, disease prognosis and mortality.[20]

### Association between obesity and periodontal disease

It has been suggested that obesity is second only to smoking as the strongest risk factor for inflammatory periodontal tissue destruction.[21] The first report on the relationship between obesity and periodontal disease appeared in 1977, when Perlstein *et al*. observed histopathologic changes in the periodontium in hereditary obese Zucker rats. Using ligature-induced periodontitis, they found alveolar bone resorption to be greater in obese animals compared with non-obese rats. Also, it seemed that under healthy oral conditions, obesity per se does not promote pathologic periodontal alterations; however, in response to bacterial plaque accumulation, periodontal inflammation and destruction were more severe in obese animals.[4] Later on, the hypothesis of obesity as a risk factor for periodontal disease was supported by epidemiological studies.

In 1998, Saito *et al*. analyzed 241 healthy Japanese individuals and showed, for the first time, an association between obesity and periodontal disease in humans. In addition, studies have indicated that the fat distribution pattern plays a crucial role in the association with periodontitis.[6,7] Another recent study by Saito *et al*. concluded that obesity is associated with deep periodontal pockets, independent of glucose tolerance status. Genco *et al*. analyzed National Health and Nutrition Examination Survey (NHANES III) data and demonstrated that BMI was positively correlated with the severity of periodontal attachment loss; they found that this relationship is modulated by insulin resistance.[8]

The underlying biological mechanisms for the association of obesity with periodontitis are not well known; however, adipose-tissue–derived cytokines and hormones may play a key role. Fat tissue is not merely a passive triglyceride reservoir of the body, but also produces a vast amount of cytokines and hormones, collectively called adipokines or adipocytokines, which in turn may modulate periodontitis.[22]

### Adipose-tissue–derived hormones and cytokines (adipokines) inflammatory markers

**Figure 1:**
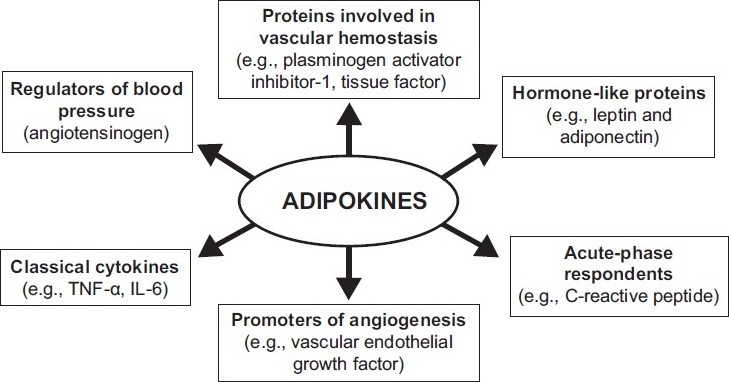
Different roles of adipokines

Adipose tissue secretes proinflammatory cytokines such as tumor necrosis factor-alpha (TNF-α) and interleukin-6 (IL-6). TNF-α and IL-6 are the main inducers of acute-phase hepatic protein production, including C-reactive protein (CRP) [Figure 1].

#### Leptin

Leptin is a pleiotropic cytokine, secreted by adipocytes, which is involved in a variety of biological processes, including energy metabolism, endocrine functions, reproduction and immunity. Leptin is thought to act as a “lipostat” that regulates adipose tissue mass. As a negative feedback mechanism, elevated leptin concentrations result in increased energy expenditure, decreased food intake and a negative energy balance. In contrast, most overweight and obese persons show resistance to leptin at the receptor level and therefore have higher leptin levels than non-overweight individuals.[16]

In addition, leptin has been shown to be involved in bone metabolism. Although reported data appear somewhat conflicting, evidence exists that leptin may decrease bone formation *via* central nervous pathways and may stimulate bone formation *via* direct peripheral effects on bone cells. The net result on bone formation may depend on various general and bone-specific factors, such as species, age, gender, serum leptin levels, blood-brain barrier permeability, bone tissue, skeletal maturity and signaling pathways.[23]

In periodontal disease, leptin regulation has still to be examined, especially with respect to the epidemiological association between obesity and periodontitis. One study implied decreasing leptin levels in gingival biopsies with increasing pocket-probing depths, which would be contrary to the cited data on other inflammatory diseases.[24]

#### Adiponectin, resistin and other adipose-tissue–derived cytokines

Adiponectin is a circulating hormone secreted by adipose tissue that is involved in glucose and lipid metabolism and which accounts for about 0.05% of total serum proteins. Contrary to other adipose-derived hormones, adiponectin levels are reduced in persons with obesity, insulin resistance or type 2 diabetes. Adiponectin improves insulin sensitivity and may have anti-atherogenic and anti-inflammatory properties, and low plasma adiponectin levels have been shown to predict type 2 diabetes and coronary heart disease in humans. Experimental models suggest that adiponectin could play a role as a mediator of inflammation; however, the exact role of adiponectin in inflammatory diseases remains to be elucidated.[25,26]

Resistin belongs to a family of resistin-like molecules (RELM) and has been reported to be secreted by adipocytes and to cause insulin resistance in animal models. However, studies have shown that the biology of resistin differs substantially between species, and many aspects, specifically its association with obesity and its effects on insulin sensitivity in humans, remain controversial. Current evidence suggests that, in humans, resistin is more closely related to inflammatory processes than to insulin resistance. Whether or not resistin plays a role in inflammatory periodontal disease remains to be defined.[27]

As more and more adipose-tissue–derived cytokines and hormones are being discovered, the complexity of the endocrine network of which these mediators are a part becomes more and more apparent. Recent additions to this list of adipokines include visfatin, which elicits insulin-like effects, and serum-retinol–binding protein 4 (RBP4). Regarded initially as markers mainly related to weight regulation and insulin resistance, it has become clear that hormones like leptin, resistin or adiponectin are involved in a variety of functions and diseases, including cardiovascular disease, diabetes and inflammatory diseases.[27]

### Association of periodontitis with obesity-related chronic diseases

**Figure 2:**
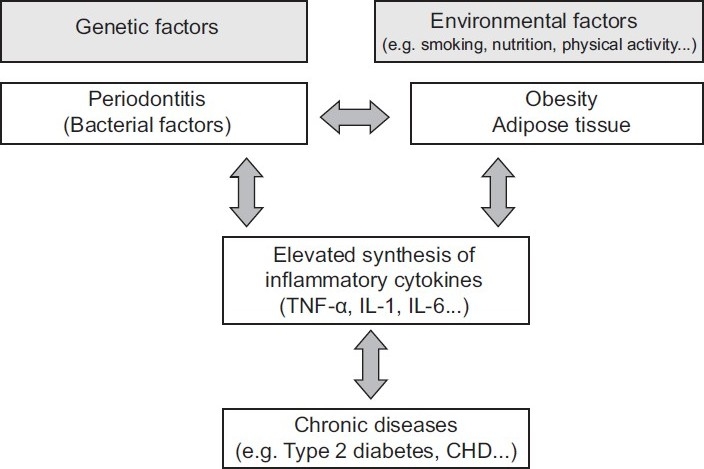
Model linking periodontitis and obesity with inflammationrelated chronic diseases

Proinflammatory cytokines may play a crucial role in the close relationship among periodontitis, obesity and chronic diseases. In fact, this association may be multidirectional [Figure 2].[28] For example, it has been well established that inflammation is an essential component in the development of atherosclerosis, and observational studies showed that periodontitis is associated with a moderately, but significantly, higher risk of coronary heart disease.[28–30] Interventional studies that examined the effects of antibiotic treatment on cardiovascular risk have generally failed to show any beneficial effect; however, these studies have mostly been of short duration (less than 1 year of treatment) and have investigated the effects on secondary prevention only. Inflammatory diseases like periodontitis induce the production of proinflammatory cytokines such as TNF-α, IL-1 and IL-6.[28] It has been suggested that the secretion of TNF-α by adipose tissue triggered by LPS from periodontal gram-negative bacteria promotes hepatic dyslipidemia and decreases insulin.

**Figure 3:**
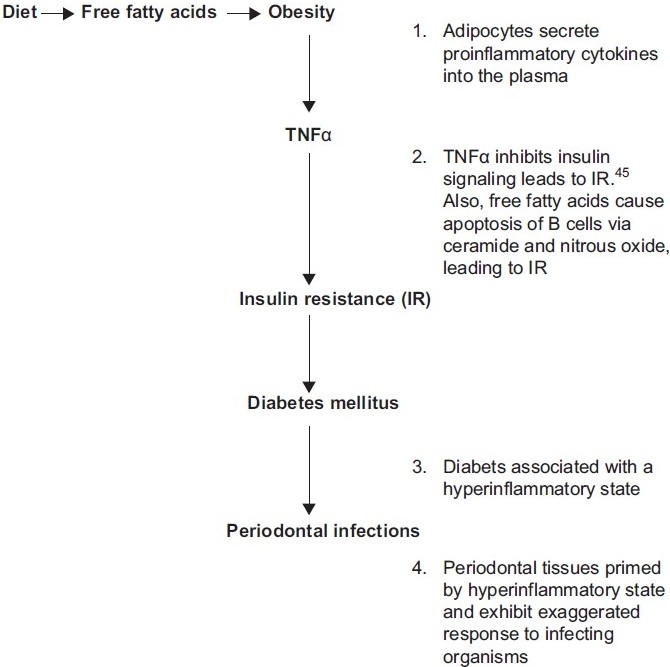
A proposed model linking inflammation to obesity, diabetes and periodontal infections

Type 2 diabetes and decreased insulin sensitivity are associated with the production of advanced glycation end products (AGE), which trigger inflammatory cytokine production, thus predisposing to inflammatory diseases such as periodontitis. These observations suggest a potential interaction among obesity, periodontitis and chronic disease incidence, although present studies are insufficient to conclude whether such associations are causal. Thus in addition to being a risk factor for type 2 diabetes and coronary heart disease, obesity-related inflammation may also promote periodontitis. Conversely, periodontitis, once it exists, may promote systemic inflammation and thereby increase the risk of coronary heart disease. In this context, it is interesting to note that periodontal treatment has been shown to reduce circulating TNF-α and serum levels of glycosylated hemoglobin, and has beneficial effects on the control of type 2 diabetes [Figure 3].[5,8,31]

### Assessment in dental practice

Until recently, a definite diagnosis of obesity was only rarely made by physicians, and body weight or body height was rarely measured in clinical practice. Further, it has been shown that about 25% of obese persons have been misclassified, by subjective estimation of the physician, as having normal weight. In future, if obesity is to be acknowledged as a multiple-risk-factor syndrome for overall and oral health, general and oral risk assessment in the dental office should include the evaluation of body mass index on a regular basis. Although there is still research ongoing as to whether BMI or waist circumference or both are a better disease risk predictor, the assessment of waist circumference in addition to BMI seems advisable, based on current obesity guidelines. Besides the suggested association between periodontal disease and obesity, periodontists need to be aware of the potential health problems related to obesity and should take them into account during treatment.[27,32]

It has been suggested that supine patient positioning should be avoided, to maximize the pulmonary mechanics. Impaired chest expansion decreases vital capacity and tidal function, which compromises tissue oxygenation. These conditions put the obese person at high anesthetic and surgical risk. Wound-healing processes are dependent on sufficient tissue oxygenation. Also, higher incidences of infections and post-surgical hematoma formation have been reported among obese persons. The vulnerability to wound complications increases morbidity and mortality of obese persons. Also, a close collaboration with the general physician and the dietician may be beneficial to ensure effective periodontal treatment.[27,32]

## CONCLUSION

Obesity is a complex disease, and its relationship to oral status has been realized by the scientific community in recent years. Periodontists must be aware of the increasing numbers of obese persons and of the significance of obesity as a multiple-risk-factor syndrome for overall and oral health. Proinflammatory cytokines may be a multidirectional link among periodontitis, obesity and other chronic diseases. The adipose tissue is a large reservoir of biologically active mediators, such as TNF-α and other adipokines. Studies have demonstrated a close involvement of the adipokines – such as leptin, resistin and adiponectin – in inflammatory processes. However, their role in periodontal inflammation has yet to be defined. Although this relationship needs further investigation, periodontists should counsel obese persons regarding the possible oral complications of obesity, to diminish morbidity for these individuals. This includes the measurement of body mass index and waist circumference for periodontal risk assessment on a regular basis.
